# Decoding the spatiotemporal dynamics of tumor immune niche remodeling in cancer immunotherapy

**DOI:** 10.3389/fimmu.2026.1827177

**Published:** 2026-05-07

**Authors:** Zhiyu Fu, Haiyan Wu, Hao Xu, Dongfeng Li, Jishun Chen, Xinwen Min, Handong Yang, Wenwen Wu, Zhixin Liu, Wei Cai, Jun Chen, Aihua Mei

**Affiliations:** 1 Sinopharm Dongfeng General Hospital (Hubei Clinical Research Center of Hypertension), Hubei Key Laboratory of Wudang Local Chinese Medicine Research, Hubei University of Medicine, Shiyan, Hubei, China; 2 Shiyan Key Laboratory of Virology, Hubei University of Medicine, Shiyan, China; 3 School of Public Health, Hubei University of Medicine, Shiyan, Hubei, China

**Keywords:** biomarkers, combinatorial therapeutic strategies, immunotherapy resistance, niche remodeling, single-cell spatiotemporal omics, tumor immune niche

## Abstract

The tumor immune niche (TIN) is a dynamic, spatially organized compartment that orchestrates immunotherapy response and resistance. Despite the clinical success of immune checkpoint inhibitors and CAR-T therapies, the TIN’s inherent immunosuppressive properties and adaptive plasticity drive significant therapeutic resistance. This review systematically decodes the spatiotemporal remodeling of the TIN-encompassing aberrant vasculature, dense extracellular matrix barriers, and metabolic competition-during various immunotherapeutic interventions. We highlight core biological processes driving this transformation, including the spatiotemporal coupling of vascular normalization with T-cell infiltration, metabolic functional switching, and myeloid cell phenotypic plasticity. Furthermore, we synthesize clinical evidence identifying molecular and spatial hallmarks of responsive versus resistant niches. Finally, we discuss how emerging single-cell spatiotemporal omics and physio-mimetic organoid models can deconstruct niche heterogeneity to inform personalized, precision combinatorial strategies for durable tumor control.

## 1 Introduction

**Figure 1:**
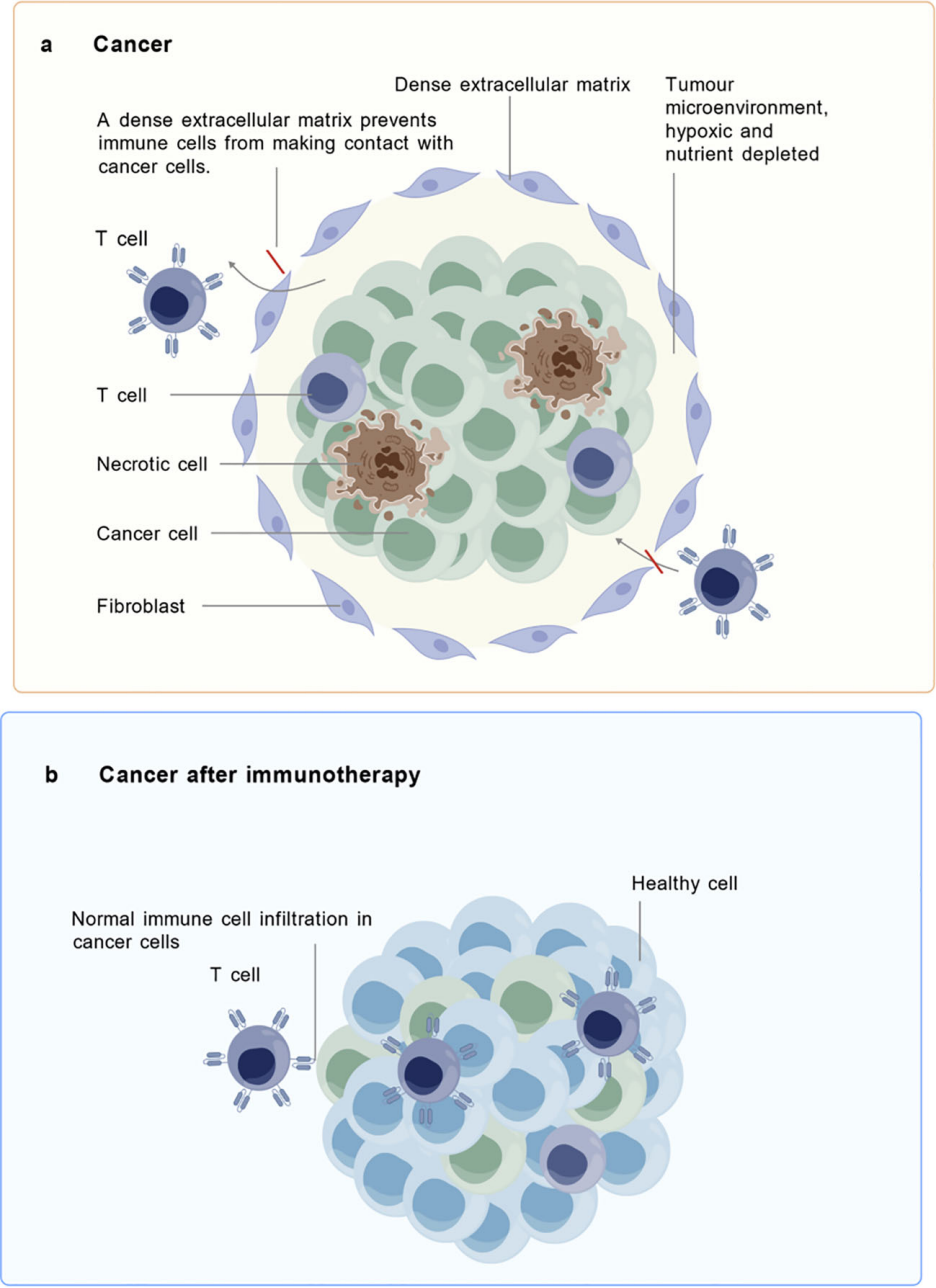
TIN before and after immunotherapy. **(A)** In the untreated TIN, cancer cells are encapsulated within a dense extracellular matrix, which impedes immune cells such as T cells from contacting them. The presence of necrotic cells and fibroblasts, along with hypoxia and nutrient deprivation, characterizes this immunosuppressive state. **(B)** following immunotherapy, TIN is remodeled, allowing for normal infiltration of immune cells which regain the ability to recognize and eliminate cancer cells, thereby restoring immune surveillance. (Created with BioGDP.com).

Tumor immune niche (TIN) shall not simply substitute for tumor microenvironment (TME) but refers to a kind of analytical model that targets on locally distributed immunity units with certain spatial organization as well as specific function and dynamical interaction. Even prior to treatment, there exist multi-dimensional abnormalities in TIN which cover some aspects such as disproportional proportion of immune cells; unusual blood vessels & hypoxic state; metabolic competition; ECM obstacles. When facing intervention like those of immune-checkpoint inhibitor, CAR-T therapy, therapeutic vaccine, cytokine-based therapy and so forth, the above mentioned axes of vascularization/hypoxia; metabolism competition; stroma/ECM obstacle; myeloid plasticity keep changing all the way through which may help effector T cell infiltrate/migrate easily, recover antigen-presenting capacity, activate immunity; while at the same time may cause T cell become exhausted, get rid away from tumor site again and even develop acquired resistance. Therefore, grasping spatiotemporally will help us understand the situation, and linking up those apparently unrelated factors can form an integrated reason; these two things are very important if we want to improve our explanation ability as well as translation efficiency (of this summary). Translational referring to previous research results, the current paper particularly emphasizes four aspects: several basic characteristics before treatment; some major bio-processes occurring following drug stimulus; certain phenotype-related proofs concerning reaction/resistance; one of the most typical pieces of translational work done on how to integrate different kinds of interventions (vascular normalization, metabolic manipulation, stroma attack, and myeloid programming), hoping that through them people could find a better theory background when designing personalized therapy plans based on the reconstruction of niche (**Figure 1**).

## 2 The conceptual framework and research significance of the tumor immune niche

### 2.1 Definition and multicomponent composition of the tumor immune niche

The tumor immune niche is defined as a dynamic and functionally distinct microenvironmental unit, constructed through a complex interplay between tumor cells, surrounding cellular components, and acellular constituents (1). Within this niche, immunosuppressive cell populations-including regulatory T-cells (Tregs), myeloid-derived suppressor cells (MDSCs), and tumor-associated macrophages (TAMs), the latter of which can comprise up to 50% of the tumor mass in many solid tumors (2). Under conditions of local immune suppression, these cells exhibit pronounced pro-tumorigenic characteristics (3).

Research indicates that the establishment of a tumor immune niche is accompanied by aberrant angiogenesis and chronic inflammation, which collectively foster an immunosuppressive microenvironment (4). Furthermore, the hallmark of the tumor immune niche is the intense competition between tumor cells and immune cells for essential nutrients such as glucose, amino acids, and lipids. This metabolic rivalry is further exacerbated by the physical barrier constituted by the extracellular matrix(ECM), which intensifies immune exclusion within the niche (5, 6). Collectively, these intricate interactions and functional specializations constitute a highly distinctive tumor immune niche, characterized by its unique cellular and molecular composition that fosters immune evasion and supports tumor progression.

### 2.2 Niche plasticity as the core determinant of immunotherapy response and resistance

TIN exhibits an inherently highly dynamic nature and remarkable plasticity, characterized by continuous cellular reprogramming and molecular adaptations that drive its functional evolution (7). Although therapies such as immune checkpoint inhibitors can effectively elicit robust clonal expansion of T cells, tumors nevertheless retain the capacity to induce T cell exhaustion, thereby facilitating immune evasion and promoting a dysfunctional state in antitumor immunity (8). This is achieved through multifaceted mechanisms, notably through the recruitment of immunosuppressive myeloid cell populations and the establishment of fibrotic physical barriers, which collectively reshape TIN to favor tumor immune evasion (9).

Therapeutic interventions that induce remodeling of TIN can yield divergent outcomes-either triggering tumor regression or paradoxically enhancing invasive and metastatic potential. This duality underscores the critical importance of elucidating the precise mechanisms through which the tumor immune niche undergoes functional and structural reprogramming during dynamic remodeling processes (10).

### 2.3 Clinical translational significance of understanding niche dynamics

**Figure 2:**
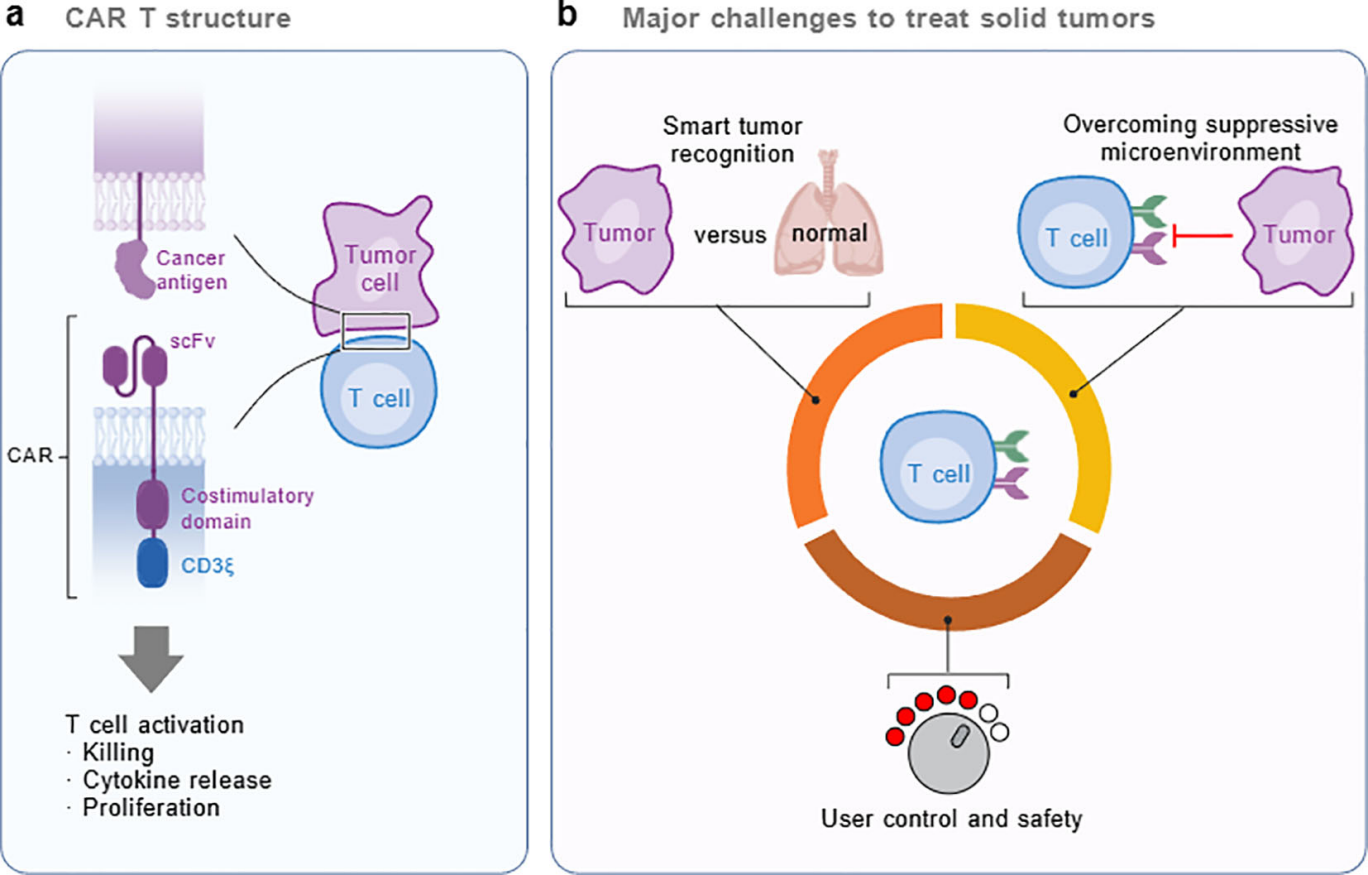
Structure of CAR-T cell and major challenges in treating solid tumors. **(A)** Schematic representation of a chimeric antigen receptor T (CAR-T) cell, highlighting key domains: the single-chain variable fragment (scFv) for antigen recognition-stimulatory domains, and the CD3 signaling domain. Upon activation, CAR-T cells mediate tumor killing, cytokine release, and proliferation. **(B)** The three major challenges in CAR-T cell therapy for solid tumors: smart tumor antigen recognition to distinguish tumors from normal tissues, overcoming the immunosuppressive TIN, and ensuring user-controllable efficacy and safety. (Created with BioGDP.com).

Elucidating the principles governing the dynamic remodeling of the tumor immune niche holds profound implications for advancing clinical translation. This significance is corroborated by observational studies revealing that metastatic lesions exhibit comprehensive reprogramming of immune cell populations and their transcriptional profiles (11). This discovery opens new therapeutic avenues for targeting metastatic disease. Building upon this foundation, novel cellular therapies such as CAR-T and CAR-macrophage treatments demonstrate significant potential to actively re-educate the immune microenvironment and provoke robust antitumor immunity (**Figure 2**).

The emerging therapeutic paradigm is evolving toward multi-pronged intervention strategies, including vascular normalization, metabolic modulation through agents like IDO inhibitors, and extracellular matrix remodeling using LOX inhibitors, all designed to synergistically overcome immune tolerance. These advances collectively underscore that translating fundamental biological insights into personalized combination therapy regimens represents the pivotal pathway to break through current limitations in cancer immunotherapy (12, 13).

## 3 Baseline characterization: pre-treatment features of the tumor immune niche

### 3.1 Composition and spatial architecture of immune cells

Within solid tumors, an immunosuppressive network predominates, comprising regulatory Tregs, MDSCs, and TAMs as its core components. Investigations in colorectal cancer reveal that infiltrating Tregs adapt to glucose-deprived conditions within TIN through the glucose-responsive transcription factor Mondo A, mediating transcriptional adaptation to metabolic stress (14). The immune infiltration profile of glioblastoma is predominantly characterized by substantial accumulation of TAMs and Tregs, which collectively establish an immunosuppressive microenvironment conducive to tumor progression (15). Within myeloid-enriched metastatic niches, a characteristic immunological shift occurs, marked by concomitant expansion of regulatory Tregs and contraction of B-cell populations, collectively reshaping the local immune landscape toward an immunosuppressive state (16, 17). Therefore, even without external perturbation, the TIN has already entered an unbalanced status –myeloid cell shows great variety and efficiency of effector T cell is limited. Then hypoxia would reshape the immune landscape and change T-cell functions’ phenotypes (18), thus enlarging aforementioned basic patterns; its mechanism will be explained in subtopic 2.2.

### 3.2 Aberrant vasculature and the formation of a hypoxic microenvironment

**Figure 3:**
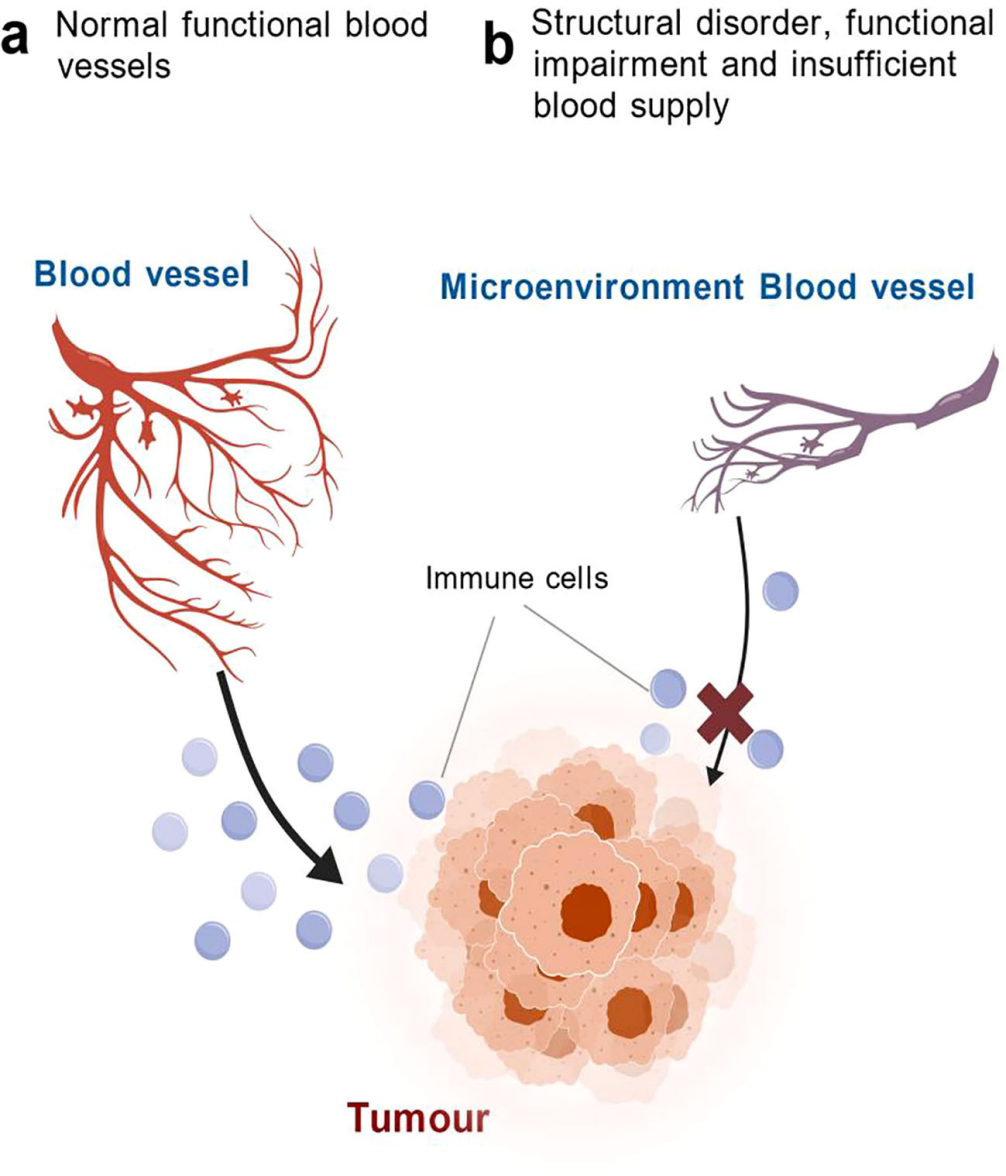
Comparison of vascular function in normal tissue versus TIN. **(a)** Normal functional vasculature is well-organized and branched, efficiently delivering immune cells to the tumor site. **(b)** In TIN, vasculature is structurally disordered and functionally impaired, leading to insufficient blood supply and markedly hindered immune cell infiltration, as indicated by the blocked arrow. (Created with BioGDP.com).

The abnormal tumor blood vessel and hypoxia are not two separate disease states, but they are the results of one disease development process that follows each other; The structural-functional abnormality of the tumor blood vessel makes part of the oxygen supply insufficient, thus forming local persistent hypoxia (19, 20). Then under this premise, the hypoxic state itself will also promote various kinds of pathological reactions in TIN immunosuppressive via different pathways (such as pathological angiogenesis, metabolism reconstruction, ECm reconstruction, Epithelial-Mesenchymal Transition, Immune Escape) (21);In addition, some factors induced by hypoxia will also be activated at this stage (such as HIF-1). These factors will continue to increase its own changes (22). In particular, the vessel-hypoxia axis connects various kinds of immunosuppressive factors which have always been talked about one by one. The disordered vessels prevent the invasion of immune cells themselves, while the hypoxia and acidosis arising from abnormal endothelial metabolism guide these cells into suppressive subsets, thus making up one kind of pathophysiological obstacle which will interfere with immune effector functions more than ever (23, 24) (**Figure 3**). Hypoxia must not be treated solely as a pathological context when studying solid tumors; instead, we need to treat it as an important node which links vascular malformation, metabolic disorder, and immunosuppression together (25).

### 3.3 The landscape of metabolic competition

Within TIN, neoplastic cells engage in metabolic reprogramming to directly compete with immune cells for limited nutrient resources, including glucose, amino acids, and lipids, thereby creating a metabolically antagonistic milieu that compromises antitumor immunity. This reprogramming not only adapts to hypoxic and acidic conditions (26, 27), but also directly suppresses immune cell function by depleting nutrients and acidifying TIN (28). Specifically, these oncogenic core metabolic pathways are altered (29) and are directly associated with the immune microenvironment, as evidenced by metabolic subtypes in hepatocellular carcinoma (30). Furthermore, tumor cells themselves can secrete signaling factors to induce metabolic reprogramming in other cell types (31). The resulting systemic state of metabolic dysregulation thereby constitutes a significant barrier to effective antitumor immune responses (32) (**Table 1**). More specifically.

**Table 1:** Key metabolic consequences in TIN.

Key metabolic	Physiological state	Abnormal changes in the TME/TIN	Major immune cells/functions affected	Potential intervention directions
Glucose/lactate	In normal tissues, supply and demand are relatively balanced and lactate levels are low	Tumor cells exhibit high glycolytic flux, preferentially consume glucose, and progressively accumulate lactate and acidity (33, 37, 39)	CD8+ T cells, NK cells, Tregs, and TAMs; suppressed cytotoxicity and enhanced suppressive polarization (33, 37)	Inhibit lactate metabolism, buffer acidosis, and combine with ICIs (33)
Tryptophan-kynurenine	Supports protein synthesis and immune homeostasis	IDO/TDO activation, tryptophan depletion, and kynurenine accumulation (33, 34)	T-cell anergy, Treg expansion, and impaired DC activation (34)	Inhibit the IDO/TDO axis; combine with biomarker-based stratification (33, 34)
Glutamine	Participates in energy metabolism and biosynthesis	Tumor and immune cells compete for glutamine, affecting metabolic adaptation (33–35)	Reduced effector T-cell function and reshaping of myeloid phenotypes (33, 34)	Redirect glutamine metabolism and combine with immunotherapy (33, 34)
Methionine/one-carbon metabolism	Supports methyl-donor availability and epigenetic homeostasis	Donor shortage or misallocation alters epigenetic programs (33, 34)	Impaired T-cell memory, differentiation, and functional stability (34)	Combined epigenetic-metabolic regulation (33, 34)
Fatty acids/cholesterol	Involved in membrane synthesis and signal transduction	Abnormal lipid uptake, lipid-droplet accumulation, and cholesterol metabolism (33, 37, 39)	Dysfunction of TAMs, exhausted T cells, and DCs (37, 39)	Regulate lipid metabolism and improve antigen presentation (33, 37)

Glucose–lactate. High glycolytic activity in tumor cells leads to excessive glucose consumption, thereby depriving effector T cells and NK cells of the energy required to maintain their homeostasis (33).

Amino acid. In TIN, the metabolism of individual amino acids leads to significantly different physiological consequences. Tryptophan depletion and kynurenine accumulation, mediated by IDO/TDO-related pathways, can lead to effector T cell anergy and expansion of Tregs (34).

Glutamine not only supports tumor growth but also influences the metabolic adaptation of myeloid cells and T cells (35). Methionine participates in methyl donor provision, epigenetic regulation, and the formation of T cell memory (36). Therefore, treating amino acid competition as a uniform, undifferentiated process would overlook the distinct effects of individual metabolites on immune regulation.

Lipids and cholesterol. Changes in fatty acid uptake, lipid peroxidation, and cholesterol metabolism are frequently linked to T cell exhaustion and TAM accumulation (37). In tumors, lipid enrichment not only serves as energy reserve but also critically determines the modes of antigen presentation and inflammatory signaling (38, 39). In hepatocellular carcinoma, the accumulation of long-chain acylcarnitines inhibits iNKT cell expansion and induces cellular senescence, thereby demonstrating how such lipid metabolites can directly restrain antitumor immunity (40).

### 3.4 The physical barrier function of the extracellular matrix

In solid tumors, dense physical barriers directly impede the infiltration of functional immune cells, thereby obstructing their cytotoxic functions. In basal cell carcinoma, cancer-associated fibroblasts (CAFs) and specific macrophage subsets contribute most significantly to the exclusion of CD8^+^ T cells and the suppression of immune activity (41). Moreover, within these highly structured immunosuppressive niches, a phenomenon exists where CAFs and macrophages establish functional connectivity through spatial proximity and intercellular communication. Their presence fosters the formation of hypoxia-induced physical barriers via extracellular matrix (ECM) remodeling, which further hampers lymphocyte infiltration into the tumor core. Together, physical barriers (immune exclusion) and metabolic suppression (nutrient competition and microenvironmental acidosis) constitute two formidable defensive shields for the tumor.

## 4 Mechanisms of immunotherapy on the Niche

### 4.1 T cell activation by immune checkpoint inhibitors

Immune checkpoint inhibitors, such as PD-1/PD-L1 blockade and CTLA-4 inhibition, can reinvigorate T-cell function (42). This therapeutic strategy induces substantial clonal expansion of tumor-specific T cells and promotes their differentiation into potent effector phenotypes (43). Furthermore, it facilitates a remodeling of the T-cell receptor repertoire, thereby enhancing the capacity of cytotoxic T lymphocytes to recognize and respond to tumor antigens (44). However, patient responses to this therapy vary significantly, a heterogeneity primarily attributable to the inherent characteristics of the patient’s immune niche prior to treatment initiation.

### 4.2 Inflammatory reprogramming induced by CAR-T cell therapy

CAR-T cell therapy can induce a potent inflammatory cytokine storm within the tumor microenvironment, characterized by massive release of IL-6, IFN-γ, and other inflammatory mediators (45). This robust inflammatory reprogramming exhibits a dual nature: while it can activate antigen-presenting cells (APCs) and enhance the intensity of antitumor immune responses (46), it may also lead to excessive T cell activation and subsequent functional exhaustion (47). Emerging evidence indicates that the intricate crosstalk between CAR-T cells and tumor-resident macrophage populations plays a crucial role in the precise modulation of the local inflammatory microenvironment. Additionally, therapy-induced alterations in vascular permeability significantly influence the infiltration efficiency of therapeutic cells into solid tumor masses, representing a key determinant of treatment efficacy (48).

### 4.3 Regulation of antigen presentation by therapeutic vaccines

Therapeutic vaccines function by modifying antigen presentation pathways within the tumor ecosystem. Their mechanism involves enhancing the capacity of dendritic cells to capture and present tumor antigens, thereby breaking the tumor-imposed suppression on antigen presentation, and inducing a specific T-cell immune response against neoantigens (49). The vaccine-induced immune response is initiated by the recruitment, expansion, or chemotaxis of priming cells in the draining lymph nodes, ultimately leading to the establishment of effector immune synapses at the tumor site. The stromal barrier and immunosuppressive microenvironment of tumors constitute the primary obstacles to achieving this antigen presentation reprogramming (50).

### 4.4 Influence of cytokines on immune cell polarization

Cytokine-based therapies can remodel the immune niche by modulating the polarization states of immune cells. For instance, IL-2 stimulates the proliferation and activation of effector T cells and NK cells, while TGF-β antagonists reverse the immunosuppressive effects of Tregs, leading to alterations in immune cell proportions and functions that favor antitumor immunity. Such interventions can create local conditions conducive to immune-mediated attack (51). However, the administration of cytokine therapies must carefully account for the inherent complexity and dual nature of cytokines, as well as the sensitivity of their efficacy to factors such as dosage, timing, and patient context (52). Stringent regulation of target selection, dosage, timing, and contextual administration is essential to mitigate potential adverse effects. Currently, clinical strategies are increasingly exploring combination therapies that integrate CAR-T cells or checkpoint inhibitors with cytokine-based approaches to achieve synergistic optimization of antitumor responses (53).

## 5 Key biological processes in niche remodeling

### 5.1 Spatiotemporal coupling of vascular normalization and immune cell infiltration

Aberrant tumor angiogenesis contributes to the establishment of an immunosuppressive microenvironment, characterized by disordered vascular architecture, endothelial dysfunction, and insufficient perfusion. Resultant hypoxia and other associated factors further promote pathological angiogenesis, malignant progression, and subsequent adaptations such as vascular metabolic reprogramming and extracellular matrix remodeling, all of which collectively foster an immunosuppressive tumor microenvironment (54). Improving the functional state of tumor vasculature can help reshape the immune milieu, a process that aligns with the degree of T cell infiltration. Temporally, vascular normalization precedes the enhancement of T cell infiltration. Spatially, the areas of normalized vasculature overlap with hotspots of CD8^+^ T cell accumulation, suggesting that vascular normalization may create physical conduits or a permissive microenvironment conducive to T cell trafficking, thereby enabling timely participation in the antitumor immune response (55).

### 5.2 Metabolic reprogramming drives functional switching in immune cells

TIN serves as the primary battlefield where immune cells and tumor cells compete for essential energy substrates such as glucose and amino acids, with this metabolic competition critically determining the efficacy of antitumor immunity. Recent investigations have elucidated how macrophages cells, and other tumor-associated immune cells undergo metabolic reprogramming to regulate their functional polarization, survival, and intercellular communication (56). Notably, PD-1 signaling serves not only as an immune checkpoint but also as a metabolic checkpoint, playing a decisive role in inducing metabolic remodeling of myeloid-derived suppressor cells while concurrently influencing iNOS-mediated type I interferon responses. Supporting this concept, ERK inhibition reduces T-cell responsiveness through mitochondrial dysfunction and metabolic rewiring, highlighting how signaling-dependent metabolic programming can reset effector states (57). Consequently, targeting the metabolic adaptability of tumor cells to reshape the immunometabolic landscape of TIN represents a crucial therapeutic strategy.

### 5.3 Stromal remodeling facilitates immune synapse formation

The dense architecture of the extracellular matrix (ECM) physically restricts interactions between immune cells and tumor cells. Studies demonstrate that actively disrupting this stromal barrier enables successful immunotherapy by initiating beneficial niche remodeling, a process involving multiple mechanisms including upregulation of matrix-degrading enzymes, chemical modifications of stromal components, and alterations in physical properties. Collectively, these changes eliminate barriers to immune synapse formation. Stromal remodeling also modulates the activation status and survival duration of immune cells, thereby directly influencing the efficacy of immunotherapeutic interventions (58). Research on pulmonary fibrosis diseases shows that different subpopulations of pericytes and fibroblasts can gather pathogenic immune cells in the local matrix microenvironment. Although this study is not cancer research, it still highlights the active role of matrix heterogeneity in shaping immune cell localization (59).

### 5.4 Phenotypic plasticity of myeloid cells regulates inflammatory status

**Figure 4:**
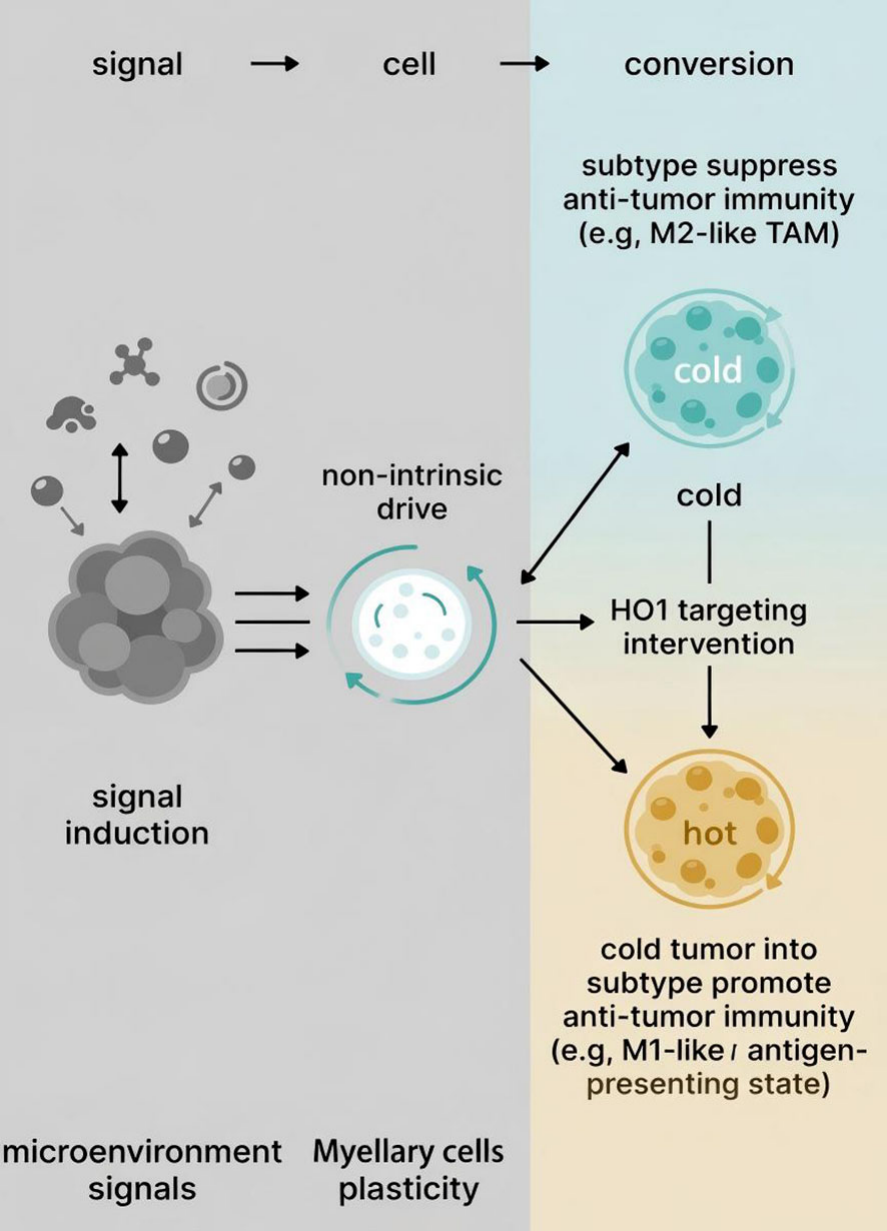
Schematic depiction of myeloid cell plasticity in TIN and its impact on antitumor immunity.

Myeloid cells exhibit a high degree of phenotypic plasticity, which is specifically induced by distinct signaling molecules-including cytokines and metabolites-within the TIN, thereby correspondingly shaping their functional polarization (60). Notably, studies demonstrate that targeted inhibition of HO-1 can drive the conversion of tumor-associated macrophages to a state that supports CD8^+^ T cell activation, effectively transitioning “cold” tumors toward an immunologically “hot” phenotype (**Figure 4**). The phenotypic reprogramming of myeloid cells fuels the dynamic evolution of the inflammatory milieu, the ultimate outcome of which depends on the predominating induced cellular subsets and the specific contextual background of the local microenvironment (61).

External signals from TIN drive the functional plasticity of myeloid cells. Without intervention, these cells may adopt an immunosuppressive phenotype (e.g., M2-like tumor-associated macrophages), contributing to a “cold” tumor state. Targeted inhibition of HO1 reprograms these cells toward an immunostimulatory phenotype (e.g., M1-like or antigen-presenting state), promoting antitumor immunity and converting the tumor into a “hot” immunologically active state. (Created with BioGDP.com)

## 6 Clinical correlations: evidence linking niche phenotypes to treatment response

### 6.1 Molecular hallmarks of a responsive niche

Tumors responsive to immune checkpoint inhibitors exhibit a characteristic molecular landscape within their immune niche. Successful treatment is reflected at the single-cell transcriptomic level by the clonal expansion of tumor-specific T cells and the maintenance of their cytotoxic effector functions (62). Transcriptomic analysis at single-cell resolution reveals that responsive microenvironments possess a coherent signature of immune activation, including the upregulation of interferon-γ signaling, enhanced antigen presentation, and secretion of pro-inflammatory cytokines (63). Concurrently, a temporal correlation is observed between elevated levels of vascular normalization signals (e.g.,Angiopoietin-2) and increased T cell infiltration (64). These features, combined with low expression of immunosuppressive metabolic enzymes such as IDO1 and CD73, constitute a favorable metabolic profile associated with positive therapeutic outcomes (65).

### 6.2 Conserved features of primarily resistant niches

Tumors exhibiting primary resistance display both spatially and functionally conserved immunosuppressive traits. Such microenvironments are typically characterized by physical immune exclusion, structurally dominated by dense extracellular matrix, and activated fibroblasts. Within this niche, MDSC and M2-polarized macrophages secrete copious amounts of immunosuppressive factors such as TGF-β and IL-10, fostering a profoundly immunosuppressive milieu (66). Metabolically, the hyperactive tryptophan-kynurenine pathway directly impairs T-cell cytotoxicity by catalyzing the production of substantial adenosine (67). Furthermore, the unique immune-privileged advantage inherent to the cancer stem cell niche within these tumors provides a foundation for sustained primary resistance.

### 6.3 Adaptive remodeling mechanisms in acquired resistance

Acquired resistance essentially represents a dynamic succession and multifaceted remodeling process driven by the continuous evolution of the tumor immune niche and complex cellular interactions under therapeutic selective pressure. Research indicates this adaptive process encompasses several key aspects: a functional shift of myeloid cells from pro-inflammatory to immunosuppressive phenotypes, abnormal accumulation of specific metabolites such as itaconate at the metabolic level, and upregulation of ECM remodeling genes including LOX family members that alter the composition and spatial distribution of physical barriers (68). These changes collectively impede immune synapse formation, ultimately driving T cells into a functionally exhausted state, and the transcriptional reprogramming of CAFs toward a pro-migratory phenotype, which under the influence of activated CAFs prevents CD8^+^ T cell infiltration into the tumor core. Together, this evidence demonstrates that acquired resistance constitutes a complex mechanism of immune escape achieved through coordinated adaptive changes across multiple layers of the tumor niche, encompassing cellular phenotypes, the metabolic milieu, and physical architecture (69).

## 7 Strategic outlook: combination therapies targeting niche remodeling

### 7.1 Combining vascular normalization therapy

Structural and functional abnormalities in tumor vasculature represent a major factor shaping the immunosuppressive microenvironment. By inducing hypoxia, these vascular defects trigger multiple downstream mechanisms-including pathological angiogenesis, metabolic reprogramming, and immune evasion that collectively undermine the efficacy of immunotherapy (70). Research indicates that vascular normalization therapy can effectively improve the functionality and structure of tumor vasculature. This therapeutic approach enhances the infiltration of CD8^+^ T cells into tumor parenchyma by augmenting hemodynamic driving forces and reducing the incidence of peripheral edema, thereby synergistically potentiating the antitumor efficacy of immune checkpoint inhibitors (71). Studies demonstrate that TGF-β3-mediated vascular normalization significantly enhances the efficacy of PD-L1 blockade therapy. The synergistic effect achieved through this combination promotes robust infiltration of CD8^+^ T cells into tumor cores. Importantly, recalibrating disordered vascular networks using anti-angiogenic agents represents a pivotal clinical strategy for improving outcomes in cancer immunotherapy (72). Recent research findings demonstrate that endothelial cell reprogramming can directly reverse the immunosuppressive vascular microenvironment in tumors, thereby overcoming existing resistance mechanisms to current immunotherapies (73).

### 7.2 Combining metabolic intervention strategies

Metabolic reprogramming within TIN serves as a critical driver of tumor-induced immunosuppression. Specifically, IDO-mediated tryptophan depletion and its product kynurenine, alongside adenosine generated via CD73 catalysis, collectively establish a profoundly immunosuppressive metabolic milieu that induces CD8^+^T cell dysfunction. Targeting this hallmark feature, IDO inhibitors can specifically block the tryptophan-kynurenine pathway, thereby reverse immunosuppression and restoring a pro-immunogenic microenvironment (74).

Concurrently, metabolic dysregulation serves as a pivotal factor driving pathological tissue remodeling. Research demonstrates that LDHA-mediated lactate metabolism contributes to vascular remodeling in pulmonary hypertension, confirming that metabolic reprogramming can induce profound pathological structural alterations (75). Furthermore, emerging evidence reveals that epitranscriptomic regulation-such as the suppression of m6A modification regulators-significant impacts immune functionality (76). These findings elucidate the multifaceted interactions between metabolism and immunity at distinct regulatory levels, thereby establishing a solid theoretical foundation for developing novel metabolic intervention strategies in combination immunotherapy (77, 78).

### 7.3 Combining stromal-targeting therapy

Dense extracellular matrix (ECM) forms a physical barrier that impedes immune cell trafficking, thereby restricting lymphocyte infiltration and contributing to tumor treatment resistance. This dynamic remodeling process involves multiple enzymatic activities, including matrix metalloproteinases (MMPs), cathepsins, and various other enzyme families that collectively regulate ECM turnover and barrier properties (79). Research demonstrates that targeted intervention in aberrant ECM structure-such as employing LOX inhibitors to block collagen cross-linking or utilizing hyaluronidase to degrade hyaluronic acid-effectively reduces interstitial pressure and enhances the penetration and distribution of both therapeutic agents and immune cells within tumor tissues (80). The regulation of mechano-metabolic crosstalk (mechano-metabolic axis) provides novel interventional strategies for stroma-targeted therapies in fibrotic and neoplastic pancreatic diseases, offering a fresh perspective for modulating pathological microenvironments through mechanical signaling pathways (81).

### 7.4 Combining myeloid cell reprogramming

The polarization of TAMs toward an immunosuppressive phenotype represents a pivotal mechanism shaping the immunosuppressive tumor microenvironment. These cells actively undermine antitumor immunity through secretion of inhibitory cytokines and production of deleterious metabolites. Previous studies indicate that activated TAMs can induce dendritic cell maturation and release chemokines such as CCL4 and MCP-1, thereby mediating the recruitment of MDSCs into peritumoral tissues and fostering an immunosuppressive milieu dominated by Tregs and MDSCs Conversely, reprogramming TAMs from an immunosuppressive to an immunostimulatory phenotype not only activates immune cells and increases local effector T cell abundance, but also modulates surface co-stimulatory molecule expression, phagocytic activity, and glycolytic pathways. This transition reduces innate immune tolerance and diminishes immunosuppressive gradients, potentially establishing M1-polarized macrophages as central coordinators of both innate and adaptive antitumor immunity (82).

Fundamental research offers novel perspectives for target discovery. Studies in obesity-related research have demonstrated that Baf60a, a key component of chromatin remodeling complexes, regulates the pro-inflammatory activity of macrophages in adipose tissue. Applying this insight to the mechanisms governing myeloid cell functional plasticity in metabolic diseases and TIN represents a potentially significant avenue for discovery (83). Subsequently, it was revealed that treatment with PPAR-γ agonists can markedly alter the stromal vascular fraction in obesity models, while also facilitating the functional reprogramming and enabling heterogeneity assessment of myeloid cells (84). These advancements, spanning from molecular mechanisms to evaluation methodologies, collectively establish a solid foundation for developing precise immunotherapeutic strategies that target myeloid cells (85).

### 7.5 Clinical translation progress of representative combination strategies

**Table 2**.

**Table 2:** Clinical translation progress of representative combination strategies of TIN remodeling.

Combination strategy	Representative regimen	Clinical phase/status	Representative findings	Implications and limitations
Vascular normalization + ICI	Atezolizumab+ bevacizumab (HCC); pembrolizumab+axitinib (RCC)	Clinically validated; standard of care for some indications	Improves OS/PFS and increases the proportion of responding patients (86, 87)	Most mature translational strategy;however,benefit varies across tumor types and niche phenotypes
Metabolic intervention+ICI (IDO1)	Epacadostat+ pembrolizumab	Phase III negative	ECHO-301 showed no improvement in PFS/OS (88)	Indicates that single-pathway inhibition cannot cover the complexity of metabolic networks; patient selection must be optimized
Metabolic intervention+ICI (CD73)	Oleclumab+ durvalumab	Benefit signal in phase II; phase III evaluation ongoing	InCOAST, ORR/PFS improved versus durvalumab alone (89)	Suggests that the adenosine axis may be a more suitable entry point for network-level metabolic intervention
Myeloid reprogramming+ICI	ATRA+ pembrolizumab (melanoma)	Phase I/II	Reduced MDSCs and showed a relatively high ORR and prolonged PFS (90)	Suitable for niches dominated by myeloid suppression, but larger validation cohorts are still needed
Stromal/stroma targeting + ICI	LOX inhibition, hyaluronan-related strategies, mechano-metabolic axis modulation	Mostly early clinical or preclinical	Current evidence mainly concerns improved infiltration and drug distribution	Potentially suitable for fibrotic/excluded niches, but excessive stromal disruption remains a risk

## 8 Current challenges: limitations and controversies

### 8.1 Technical limitations in dynamically monitoring niche heterogeneity

A paramount challenge in current research involves accurately and effectively monitoring the dynamic tumor immune niche. Single-cell transcriptomic studies reveal significant inter-patient heterogeneity even within the same tumor type. Spatial transcriptomic analyses further demonstrate that cancer-associated fibroblasts (CAFs) and specific macrophage subtypes can organize into spatially distinct immunosuppressive compartments, actively excluding CD8^+^ T cells to the tumor periphery. While current molecular imaging technologies provide valuable insights into systemic immune activation states (91). They remain insufficient for resolving the spatial architecture of intercellular interactions or bridging microscopic cellular events with macroscopic clinical manifestations. This critical gap underscores the urgent need for multimodal approaches that integrate high-resolution spatial biology with dynamic functional imaging to decode the complex topology of immune evasion mechanisms (92).

### 8.2 Balancing systemic and local immune responses

Systemic immune activation represents a potent strategy in antitumor immunotherapy, exemplified by STING pathway agonists that have demonstrated promising efficacy in preclinical studies. However, the robust inflammatory response they induce may inadvertently promote tumor immune evasion, and systemic administration carries the risk of triggering severe systemic inflammatory syndrome. Conversely, excessive local immune activation can precipitate cytokine release syndrome within TIN. Similarly, approaches such as IL-12-engineered myeloid cells (IL12-GEMys) can effectively reverse the immunosuppressive state of the pre-metastatic niche, though their comprehensive systemic impact remains to be fully elucidated. Nanoparticle-based platforms can be designed to provide multimodal stimulation to dendritic cells, thereby prolonging adaptive immune responses (93). However, the heterogeneous targeting specificity to different tumor sites following systemic administration significantly influences their overall therapeutic efficacy (94).

### 8.3 Limited predictive value of preclinical models

Current preclinical models possess significant limitations. For instance, the vasculature generated in humanized mouse models differs from that in humans, failing to accurately recapitulate the complete process of immune cell infiltration. Secondly, organoid models lack a fully functional microenvironment for cell migration, thus unable to genuinely reflect intercellular interactions (95).

Spatial heterogeneity is also remarkably pronounced across various tumor types. For example, specific microenvironmental architectures identified in single-cell analyses of basal cell carcinoma cannot be replicated in mouse xenograft models (96). While previously investigated novel approaches, including sialic acid-targeted therapies, have shown promise in preclinical studies (97), data on the extent of their remodeling effects in humans remain limited (98). Consequently, developing organoid-immune co-culture models that better preserve the spatial architecture and cellular composition of primary tumors is of paramount importance (99).

## 9 Future perspectives: research directions and technological advancements

### 9.1 Application of single-cell spatiotemporal omics technologies

Single-cell spatiotemporal omics offers a novel and powerful approach for deconstructing the dynamic evolution of the tumor immune niche (100). This technology enables the precise tracking of T-cell clonal dynamics-including expansion, migration, and exhaustion in the context of immunotherapy (101), thereby capturing the intricate spatiotemporal heterogeneity of tumor-immune features (102). Building on this, spatiotemporal omics can be leveraged to identify therapy-responsive cellular subsets and their regulatory networks, laying the groundwork for developing novel combination immunotherapeutic strategies.

### 9.2 Development of organoid-immune co-culture models

The organoid-immune cell co-culture system can closely recapitulate the *in vivo* state of bidirectional tumor-immune signaling (103). The organoid-immune cell co-culture system can closely recapitulate the *in vivo* state of bidirectional tumor-immune signaling. This technology also enables the simultaneous identification of intrinsic immune evasion properties harbored by tumor cells. Acoustic virtual 3D scaffolding technology facilitates direct contact between tumor organoids and immune cells in the absence of physical barriers, allowing for detailed analysis of their interplay. Vascularized pancreatic ductal adenocarcinoma (PDAC) organoid models have demonstrated that the vascular niche can maintain cancer stem cell properties (104), making such models highly valuable for investigating the molecular mechanisms by which immunotherapy induces changes in the vascular niche.

### 9.3 Optimization of personalized combination therapies

Drug screening platforms based on patient-derived organoids co-cultured with immune cells provide a high-throughput tool for evaluating inter-individual efficacy differences of therapies such as immune checkpoint inhibitors and CAR-based regimens (105). Furthermore, employing single-cell RNA sequencing to compare complete responders with partial responders facilitates the design of rational combination therapies at the molecular level (106). Current research directions are increasingly focused on developing strategies that simultaneously target the vascular niche and immune checkpoints, for example, the combination of anti-angiogenic agents with PD-1/PD-L1 antibodies (107, 108), exploring the synergistic effects of metabolic intervention and myeloid cell reprogramming, such as combining IDO inhibitors with CSF-1R inhibitors (109) and optimizing stroma-targeting therapies combined with immunotherapy, exemplified by LOX inhibitors paired with T-cell activating treatments (110).

To achieve optimal therapeutic outcomes, these approaches must be integrated with patient-specific organoid drug sensitivity data and relevant immunological profiling. The goal is to identify reliable biomarkers that can predict prognosis and to guide personalized, precise treatment strategies based on the molecular features of the individual’s tumor immune niche.

## 10 Summary and key conclusions

**Figure 5:**
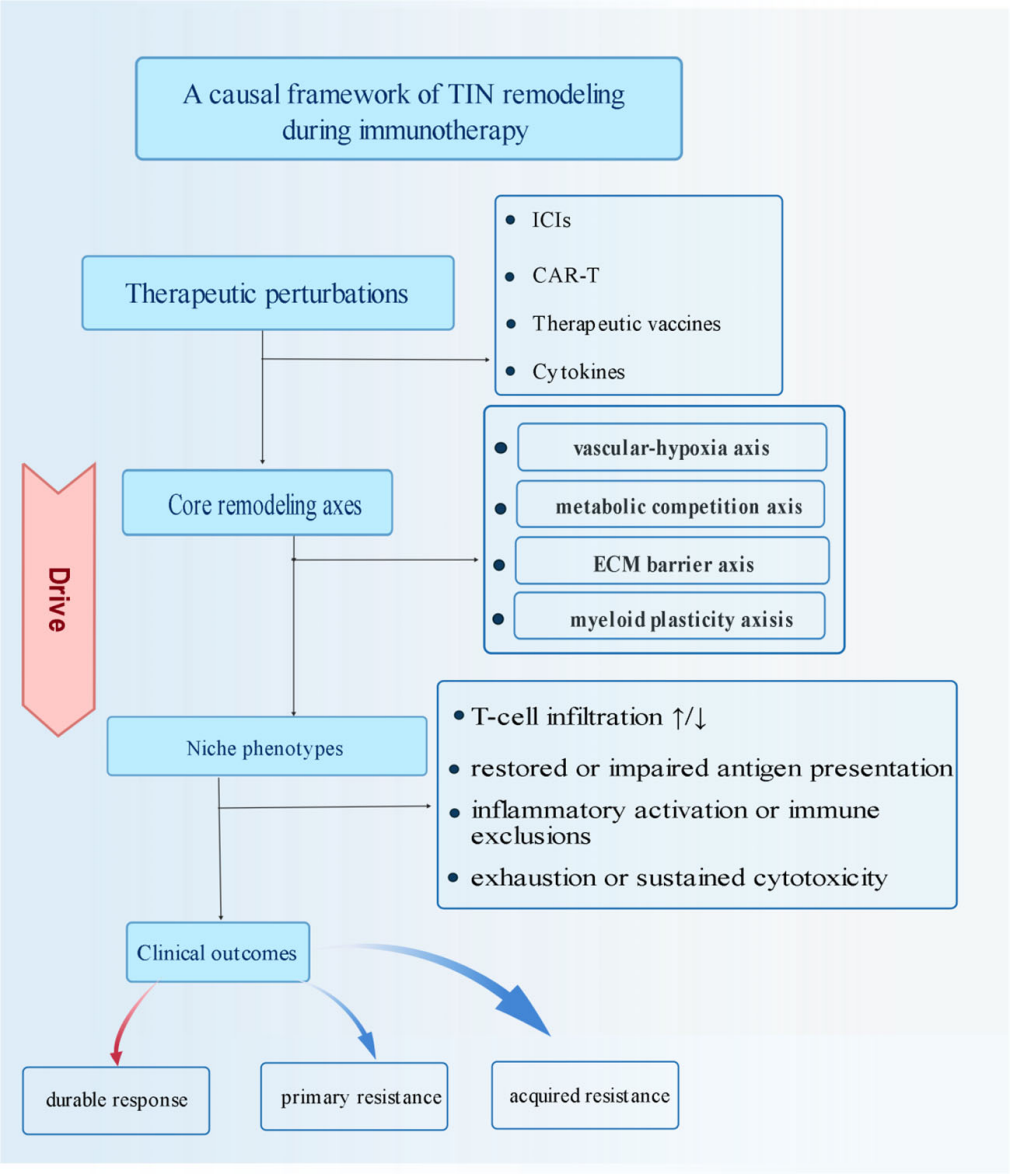
A causal framework of TIN remodeling during immunotherapy.

Elucidating the dynamic remodeling mechanisms of the tumor immune niche is instrumental for comprehensively deciphering the patterns of immunotherapy response and resistance.​This review systematically synthesizes the multidimensional and multiscale spatiotemporal succession within TIN during immunotherapy, revealing the relationship between niche remodeling and clinical efficacy (**Figure 5**). A comparative summary indicates that Immune Checkpoint Inhibitors (ICIs) primarily reverse the immunosuppressive balance by releasing the brakes on T cells (111), whereas CAR-T therapy aims to eliminate tumor cells by inciting a localized inflammatory storm, reshaping the landscape of the individual’s tumor microenvironment, and restoring the body’s intrinsic immune surveillance capacity.​The spatiotemporal coupling of vascular normalization and T-cell infiltration the metabolic reprogramming-mediated functional shift of immune cells (112), and the phenotypic plasticity of myeloid cells​constitute three core dynamic processes influencing therapeutic outcomes. Niche-targeting combination therapies have demonstrated promising efficacy. Among these, the combination of vascular normalization agents with ICIs most significantly enhances T-cell infiltration. Another prominent strategy involves targeting formidable physical barriers, such as using LOX inhibitors​against cross-linked extracellular matrix enzymes. Furthermore, combining CSF-1R inhibitors (targeting myeloid cell reprogramming) with immunotherapy may represent a breakthrough in overcoming resistance. It is foreseeable that personalized combination regimens will positively impact treatment efficacy, although several questions remain to be explored (113).

However, current challenges lie in the technological bottleneck for dynamically monitoring the highly heterogeneous niche, balancing systemic immune activation with local microenvironmental remodeling (114), and bridging the gap between preclinical models and the authentic human microenvironment-key scientific issues demanding resolution.

Future research should focus on: advancing single-cell spatiotemporal omics​to deconvolute niche dynamics; establishing more physio mimetic organoid-immune co-culture systems; leveraging artificial intelligence to optimize combination strategies (115), and a deeper understanding is still needed of the central mechanistic roles played by metabolic reprogramming and epigenetic regulation in the dynamic remodeling of the niche (116, 117). From a translational perspective, the combination of vascular normalization and immunotherapy is currently supported by the strongest clinical evidence. Metabolic intervention, in turn, suggests that therapeutic strategies must move beyond single-pathway inhibition toward network-level remodeling. By comparison, stromal targeting and myeloid reprogramming may be better suited as precision combination modules for specific TIN phenotypes. Looking ahead, the key task is not merely to further describe the composition of the niche, but to truly bridge “which niche is present” with “which combination strategy should be applied,” using approaches such as single-cell spatiotemporal omics, organoid–immune co-culture systems, and multimodal biomarker stratification. Through multi-omics integration and precise modulation of niche states, such efforts may ultimately improve the efficacy of immunotherapy and advance cancer treatment toward a more individualized therapeutic paradigm.
